# Spiritual Intelligence and High Risk Behaviors

**DOI:** 10.5812/ijhrba.18477

**Published:** 2014-02-15

**Authors:** Sedigheh Moallemi

**Affiliations:** 1 Department of Deputy, Zahedan University of Medical Sciences, Zahedan, IR Iran

**Keywords:** Intelligence, Spirituality, Spiritual Therapies

With each passing day, more people believe in spirituality as the key solution to all psychological and social problems; similarly, more experts concentrate on supporting spiritual beliefs and behaviors as a solution to these problems (1).

In this new era, experts perceive the necessity of discussing the issue of spirituality and religion from various points. Humans’ need for spirituality emanates from their nature. Although during the Renaissance, some historical events led to man’s separation from spirituality, by the beginning of the mid-20^th^century, such a need emerged again, to the extent that in 1980, some said, “God is coming back!”

Of course several factors contributed to this return, including the two worldwide wars which made man worried and pessimistic about scientific developments technology development dose not led to sincere relationships between people, but also unbelievably extended man’s relationship with organizations and places make a great shock to the modern life. Such worries and unpleasant, offensive instances have inspired mankind to make a serious decision about his resort to spirituality (2).

In this regard, the World Health Organization (WHO) has recently defined man as a psychological, social and spiritual creature. Considering the new spiritual orientation, its relationship with religion, and also other psychological issues like mental health, some experts have intended to introduce new concepts into the religion and spirituality. Thus, on the threshold of the third millennium, concepts like spiritual health, spiritual welfare, and the like, have been added to the academic literature of the psychology (3).

Recently, the psychologists have introduced a new factor called the spiritual intelligence (3) as an effective factor on mental health, which has attracted worldwide attention and interest. Spiritual intelligence represents a collection of spiritual capabilities, capacities and resources leading to the increase of people’s mental health and adaptability (4, 5). Due to the novelty of spiritual intelligence, few empirical researches have been carried out in this field so far.

Spiritual intelligence applies spiritual data to solve daily problems and provides the person`s compliance with the situation (6). Vaughan believes that spiritual intelligence unites the spiritual, inner life and the outer life (workplace). With reference to his study verifying the relationship between the spiritual intelligence and the decrease of workplace problems (8), we can conclude that spiritual intelligence improves the quality of life. In his point of view, spiritual intelligence is essential for the recognition of the choices effective on human`s psychological welfare and health improvements (7). Other studies showed a positive relationship between spiritual intelligence and features like character trait, social sensitivity, life satisfaction, energy and activity (8, 9).

Spiritual intelligence is regarded as a way of processing personal experiment and insight (5). We may conclude that spiritual intelligence affects data processing and classification in some specific scheme. Spiritual intelligence is capable of forming and organizing our perceptions about some notions, including health and disease. Spiritual intelligence improves not only personal health and welfare, but also helps people to tolerate difficult experiences such as grief and loss (7).

Some researchers consider the effectiveness of spiritual intelligence on man’s life even beyond those issues indicated above. For instance, Zohar believes that spiritual intelligence is a basis for the better effectiveness of the rational and emotional intelligences (5). Emmons indicates that spiritual intelligence may be considered as a rational understanding of the world (6).

Now, we mention some of the problems and difficulties that the 21^st^ century man is struggling with. The extent of man’s problems is increased day after day around the world, and this fact reminds us of the necessity of spirituality.

In human history, about 191 million people have lost their lives either directly or indirectly because of violence, while more than half of them have been civilians. In 2000, about 300,000 people were killed due to direct violence. Reference to an estimate in 2000, about 199,000 youngsters, in addition to 565 infants had been killed around the world (10). Also, since the beginning of 2014, about 157,057 people committed suicide (11).

According to the annual report of WHO in 2008, about 200 million people had been addicted to drugs all over the world. Despite the lack of accurate statistics on the number of addicts in our country, state officials have reported that number to be more than two million. In most countries, the vulnerability age to addiction has been around 20 to 34 years old (12). Other critical problems have been caused by drinking alcohol. Although alcohol consumption is forbidden in most religions, it has been increasingly consumed even in Islamic countries (13). All over the world 366,316 dead have been reported due to the alcohol consumption since the beginning of 2014 (11).

The researchers estimate that 35% of violence in the US is alcohol-related. Up to 70 percent of the alcohols addicted commit suicide. On the whole, alcohol is the cause of 4 percent of death worldwide annually. Daily, the average 565 of the 10-29 year old persons are murdered in result of interpersonal violences (10). Those issues affect both adults and infants. According to the international report of violence against children during 2002, about 150 million girls and 73 million boys had been sexually abused. Among 218 million working children in 2004, 5.8 million had been forcibly employed; 1.8 million forced into prostitution; and 1.2 million had been victims of human trafficking (10). And eventually there is the unfortunate tragedy of rape; the actual range of rape is not definite, however with reference to the data, one in four women in the world has been sexually abused at least once in her lifetime (10).

Spirituality has been increasingly identified as an important resource for coping with crisis and trauma. Spirituality is the source of protecting the survivors of trauma from its adverse consequences. During a study carried out by 18 researches, 17 showed a relationship between spirituality and some sexual decisions like delaying first sex, the number of sex partners and the application of contraception (14).

Despite sufficient empirical support of positive relationship between spiritual intelligence and health criteria (15-19), many aspects of this relationship have remained unknown. The results of a research showed that spiritual intelligence has a significant relationship with the quality of life together with all its subscales (physical, psychological, social and environmental) and also there is a significant and positive relationship between resiliency and the quality of life together with all its subscales (16).

Generation of a personal meaning (the component of spiritual intelligence) makes it possible that an individual creates a new situation, even stressful and worrisome, in which he finds a meaning or purpose which first helps individual to become adapted to the new condition, and then to change the stressors and eventually to reduce their negative effects. Similarly, the generation of a personal meaning while facing a hard situation, would guide the person to a meaning – centered solution and function as a problem solving method (20).

Zohar and Marshal described the characteristics of the people enjoying high spiritual intelligence as the following: they are flexible; enjoy a high level of awareness toward themselves; are capable of facing problems and pains; and find a solution to overcome hardships. Other characteristics of these people include receiving inspiration from values and insights, refusing to harm anybody, monism thinking (realizing the relationship between objects and various phenomena), searching for the answer of fundamental questions, independence and resistance to the society’s common methods and traditions. It can be said to spiritual intelligence includes having a sense of meaning, a spiritual mission in life, a sense of sanctity in life, balanced perception of the value of material and at last believing in the world`s better future (16).

Words and images asthe resources of love and support, reduce stress and exert a beneficial effect on specific mechanisms of body and probably increase the components of the spiritual intelligence resiliency by the same mechanism (21); thus, spiritual intelligence leads us to change or to improve specific situations and, indeed, to handle various circumstances (8).

With regard to the above-mentioned items, it is necessary to design some treatments and evaluate them based on the components of spiritual intelligence, in accordance with social – cultural situations and the type of disorder. Since this item is useful both for prevention and improvement of the health level, it’s deserving that the researchers and therapists explore this area more than ever.
